# Nebulized antibiotics for ventilator-associated pneumonia: a systematic review and meta-analysis

**DOI:** 10.1186/s13054-015-0868-y

**Published:** 2015-04-07

**Authors:** Fernando G Zampieri, Antonio P Nassar Jr, Dimitri Gusmao-Flores, Leandro U Taniguchi, Antoni Torres, Otavio T Ranzani

**Affiliations:** Cooperative Network for Research - AMIB-Net, Associação de Medicina Intensiva Brasileira, São Paulo, Brazil; Emergency Medicine Discipline, Faculty of Medicine, University of São Paulo, São Paulo, Brazil; Intensive Care Unit, Hospital Alemão Oswaldo Cruz, São Paulo, Brazil; Adult Intensive Care Unit, A.C. Camargo Cancer Center, São Paulo, Brazil; Intensive Care Unit, University Hospital Prof. Edgar Santos, Universidade Federal da Bahia, Rua Augusto Viana, Salvador, 40110-910 Brazil; Programa de Pós-graduação em Medicina e Saúde (PPgMS) - Faculdade de Medicina da Bahia, Universidade Federal da Bahia, Salvador, Brazil; Research and Education Institute (IEP), Hospital Sirio-Libanes, São Paulo, Brazil; Institut Clinic de Pneumologia i Cirurgia Toràcica, Servei de Pneumologia, UVIR, Universitat de Barcelona, IDIBAPS, CIBERES, Barcelona, Spain; Amil Critical Care Group, Hospital Paulistano, São Paulo, Brazil; Respiratory Intensive Care Unit, Pulmonary Division, Heart Institute, Hospital das Clínicas, University of São Paulo, São Paulo, Brazil

## Abstract

**Introduction:**

Nebulized antibiotics are a promising new treatment option for ventilator-associated pneumonia. However, more evidence of the benefit of this therapy is required.

**Methods:**

The Medline, Scopus, EMBASE, Biological Abstracts, CAB Abstracts, Food Science and Technology Abstracts, CENTRAL, Scielo and Lilacs databases were searched to identify randomized controlled trials or matched observational studies that compared nebulized antibiotics with or without intravenous antibiotics to intravenous antibiotics alone for ventilator-associated pneumonia treatment. Two reviewers independently collected data and assessed outcomes and risk of bias. The primary outcome was clinical cure. Secondary outcomes were microbiological cure, ICU and hospital mortality, duration of mechanical ventilation, ICU length of stay and adverse events. A mixed-effect model meta-analysis was performed. Trial sequential analysis was used for the main outcome of interest.

**Results:**

Twelve studies were analyzed, including six randomized controlled trials. For the main outcome analysis, 812 patients were included. Nebulized antibiotics were associated with higher rates of clinical cure (risk ratio (RR) = 1.23; 95% confidence interval (CI), 1.05 to 1.43; I^2^ = 34%; D^2^ = 45%). Nebulized antibiotics were not associated with microbiological cure (RR = 1.24; 95% CI, 0.95 to 1.62; I^2^ = 62.5), mortality (RR = 0.90; CI 95%, 0.76 to 1.08; I^2^ = 0%), duration of mechanical ventilation (standardized mean difference = −0.10 days; 95% CI, −1.22 to 1.00; I^2^ = 96.5%), ICU length of stay (standardized mean difference = 0.14 days; 95% CI, −0.46 to 0.73; I^2^ = 89.2%) or renal toxicity (RR = 1.05; 95% CI, 0.70 to 1.57; I^2^ = 15.6%). Regarding the primary outcome, the number of patients included was below the information size required for a definitive conclusion by trial sequential analysis; therefore, our results regarding this parameter are inconclusive.

**Conclusions:**

Nebulized antibiotics seem to be associated with higher rates of clinical cure in the treatment of ventilator-associated pneumonia. However, the apparent benefit in the clinical cure rate observed by traditional meta-analysis does not persist after trial sequential analysis. Additional high-quality studies on this subject are highly warranted.

**Trial registration number:**

CRD42014009116. Registered 29 March 2014

**Electronic supplementary material:**

The online version of this article (doi:10.1186/s13054-015-0868-y) contains supplementary material, which is available to authorized users.

## Introduction

Ventilator-associated pneumonia (VAP) is an important infection that develops in approximately one-third of patients who are mechanically ventilated for more than 48 hours [1,2]. VAP has caused great concern for physicians and managers because it is associated with high morbidity, mortality [3] and healthcare system costs [4]. One of the cornerstones of VAP management is antibiotic treatment, which currently presents a major challenge because of the emergence of resistant pathogens, a lack of new drugs and high associated costs.

Nebulized antibiotics have been used to treat respiratory tract infections for the last 70 years [5,6]. Many theoretical advantages of nebulized antibiotic therapy have been proposed, such as higher drug levels at the infection site and fewer systemic side effects [7]. These potential benefits would therefore enhance the antimicrobial therapy and reduce adverse effects [8]. However, clinical and technical issues regarding the use of nebulized antibiotics, primarily the best approach to deliver the drug to the lungs, have become barriers to the proper study of this technique [7-11].

There has been a resurgence in interest in this type of antibiotic administration in recent years because of the appearance of multidrug-resistant (MDR) pathogens [8,12]. Relapse and recurrence after initial treatment are also common, and monotherapy with nebulized antibiotics could be an alternative treatment. It is difficult to achieve microbiological eradication for certain pathogens, including MDR pathogens in VAP [13]. Because MDR pathogens are frequently only susceptible to older antibiotics associated with significant side effects (such as renal failure), nebulized therapy represents an interesting approach to decrease the toxicity of these drugs in critically ill patients. Nevertheless, with the exception of colistin, there are no experimental data that support the idea that nebulized antibiotics reduce systemic toxicity [11]. Adjunctive combined therapy (inhaled and intravenous) has also been suggested to maximize therapy [14]. Clinicians have a positive view of nebulized antibiotics; in a recent survey, 70% of physicians reported that adjunctive nebulized antibiotics could increase the effectiveness of VAP treatment [6].

Therefore, we sought to review the currently available evidence regarding the use of nebulized antibiotics for VAP treatment because there is no evidence supporting their use in the clinical practice. Studies that compared the use of nebulized antibiotics, with or without systemic (intravenous) therapy, with systemic therapy alone were included. We hypothesized that nebulized antibiotics would improve the clinical response success rate in the treatment of VAP.

## Methods

### Literature search

Studies were identified through a standardized search of Medline (via OvidSP), Scopus, CENTRAL (Cochrane Central Register of Controlled Trials), EMBASE, Biological Abstracts, CAB Abstracts, Food Science and Technology Abstracts (via OvidSP), Lilacs (*Literatura Latino-Americana e do Caribe em Ciências da Saúde*) and Scielo (*Scientific Electronic Library Online*) databases. A sensitive search strategy was used, which combined the following keywords: “ventilator associated pneumonia”, “VAP”, “hospital acquired pneumonia”, “HAP” or “nosocomial pneumonia” and “inhaled”, “inhalation”, “aerolized”, “aerolised”, “nebulized” or “nebulised”. The references in the included studies and personal files were also searched. The search strategy was restricted to randomized clinical trials and observational studies with matched groups (cohort studies with comparable groups or case–control studies) performed in adult subjects and published prior to 29 March 2014. We excluded care series and reports. There was no language restriction. The titles and abstracts were assessed for eligibility, and full-text copies of all of the articles deemed potentially relevant were retrieved. A standardized eligibility assessment was independently performed by two reviewers (APN and FGZ). We selected the larger study in cases of studies that reported data in more than one publication. Disagreements were resolved by consensus.

The PRISMA statement was used for guidance [15], and the meta-analysis was registered in the PROSPERO database (CRD42014009116). Ethical approval was not required for this work.

### Study selection

Studies that fulfilled the following criteria were included: 1) compared nebulized antibiotics with or without intravenous antibiotics with intravenous antibiotics only in the treatment of patients with an established diagnosis of VAP; and 2) reported at least one of the following outcomes: clinical cure, microbiological cure, mortality, mechanical ventilation duration and ICU length of stay.

### Data extraction and quality assessment

A data extraction sheet was developed. Two authors (APN and FGZ) independently extracted the following data from the included studies: year of publication, country, study design, number of patients designated to nebulized or intravenous-only antibiotics, microbiological cure criteria, clinical cure criteria, severity score, mechanical ventilation duration, ICU length of stay, microbiological and clinical cure rates, mortality and adverse events. The authors of the included studies were contacted by email to complete the missing data that were required for study characterization. A maximum of four contacts was attempted per study over a period of 6 months.

Two of the authors (APN and FGZ) assessed the risk of bias in the individual trials using the Cochrane risk of bias tool [16] for randomized clinical trials and the Newcastle-Ottawa Scale for cohort and case–control studies [17]. For the outcomes in each of the included randomized clinical trials, the risk of bias was reported as “low risk”, “unclear risk”, or “high risk” in the following domains, according to the Cochrane risk of bias tool: random sequence generation; allocation concealment; blinding of participants and personnel; blinding of outcome assessment; incomplete outcome data; selective reporting; or other bias. Disagreements were resolved by consensus with a third author (OTR).

### Outcome measurements

The primary outcome was clinical cure, as it was defined by each study author. The secondary outcomes were microbiological cure, mortality (considering the longest follow-up reported by the authors), ICU length of stay, duration of mechanical ventilation and adverse events.

### Statistical analysis

A random-effects (DerSimonian-Laird) model was employed because of the anticipated variability between the trials regarding patient samples and medical interventions. A constant continuity correction of 0.5 was used for handling zero-event studies to include all selected studies in the analysis and minimize bias. The differences observed between the treatment groups are expressed as the relative risk (RR) for categorical variables and the standardized mean differences (SMDs) for continuous variables, both with 95% confidence intervals (CIs). Heterogeneity was assessed by the I^2^ statistic and was classified as low (<25%), moderate (25 to 50%) or high (>50%). We also used funnel plots for the main outcomes in order to assess publication bias. An *a priori* subgroup analysis was performed separately analyzing randomized controlled trials and observational studies for all outcome measurements. We explored heterogeneity for study characteristics (randomized controlled trials versus observational studies) with meta-regression for the primary outcome. We assessed *post hoc* if using a different method for random-effects (Biggerstaff-Tweedie) for the main outcome would produce different results. Biggerstaff-Tweedie may be the most appropriate method when only a limited number of studies are available [18].

A trial sequential analysis (TSA) was performed using the required information size to construct sequential monitoring boundaries. The boundaries were established to limit the global type error to 5% and were calculated with the O’Brien-Fleming function, which considered a power of 80% to detect a 20% increase in clinical cure rate and a 37.5% incidence of failure of VAP treatment, as suggested by a recent meta-analysis [19]. The heterogeneity for information size calculation was set using the D^2^ measure. D^2^ is the relative variance reduction when the model is changed from a random-effects to a fixed-effect model, and its interpretation is similar to that of I^2^ because it is a proportion. However, it is advisable to use D^2^ instead of I^2^ for the required sample size information [20]. All of the observational studies were included in the TSA as high bias. The analyses were performed using R project software, version 3.1.1, with R Studio, version 0.98.1049, and the meta package (version 3.1-1) by Guido Schwazer (http://cran.r-project.org/web/packages/meta/meta.pdf). TSA was performed using TSA software, version 0.9 beta (Copenhagen Trial Unit, Copenhagen, Denmark).

## Results

### Study characteristics

**Figure 1:**
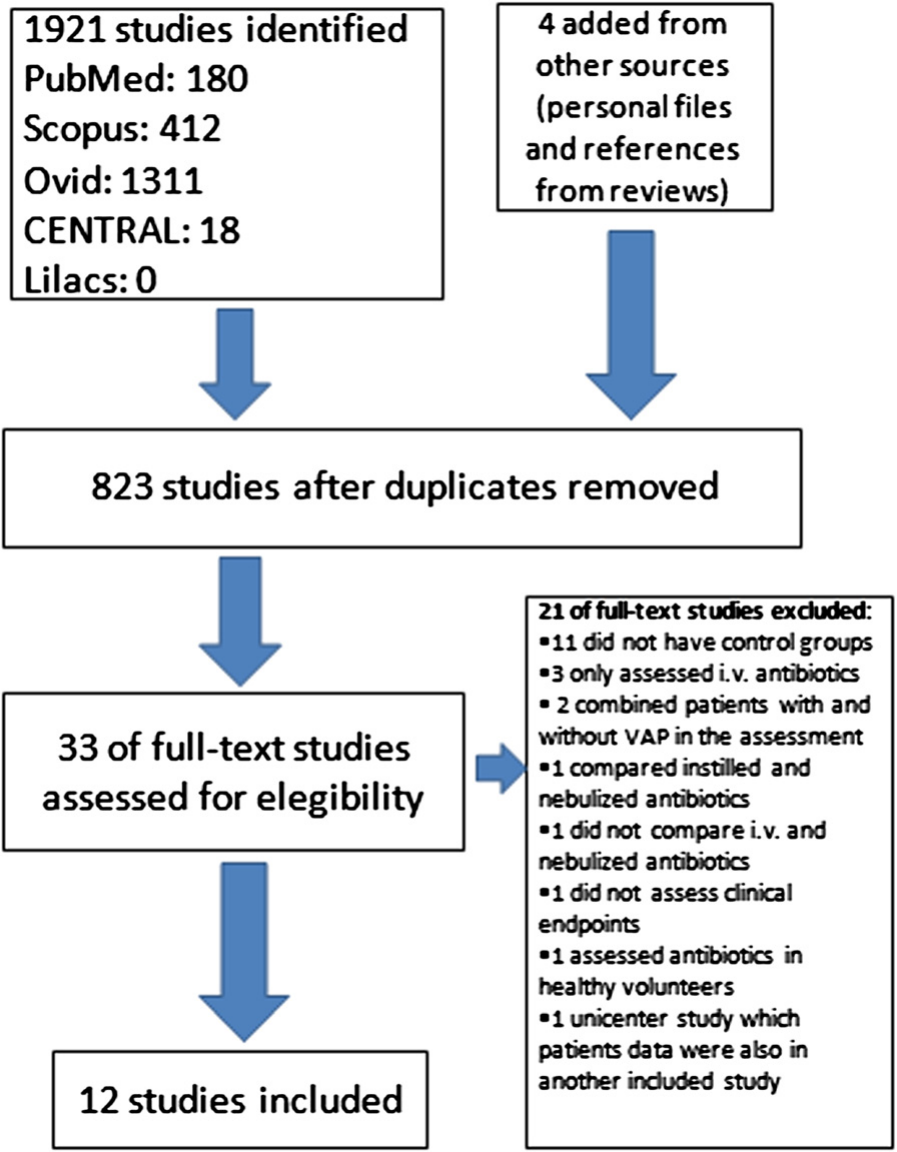
**Study flowchart.** i.v., intravenous; VAP, ventilator-associated pneumonia.

Of the 1,921 references initially identified, 33 full-text articles were assessed for eligibility, and 12 studies were selected for the analysis [21-32] (Figure 1).

Table 1 summarizes the details of the included studies. There were six observational studies and six randomized clinical trials. Two randomized controlled trials and one observational study were multicenter studies [21,26,28]. *Acinetobacter* spp., *Pseudomonas* spp. and *Klebsiella* spp. were the most isolated bacteria. The most common nebulized antibiotics administered were colistin (nine studies) and aminoglycosides (seven studies). Eleven studies compared adjunctive nebulized antibiotics with intravenous antibiotics, and only one compared nebulized antibiotics alone with intravenous antibiotics [27].

**Table 1:** **Study characteristics and quality assessment**

**Study, year**	**Country**	**Number of patients (inhaled ± intravenous antibiotic/intravenous only)**	**Isolated bacteria (n)**	**Nebulizer device**	**Inhaled antibiotic given (daily dose)**	**Quality assessment**
**Observational studies**						Newcastle-Ottawa Scale
Doshi, 2013 [21]	USA	44/51	*Acinetobacter* (61), *Pseudomonas* (53), ESBL Enterobacteria (11)	Jet or vibrating mesh nebulizer	Colistin 150-300 mg	7
Ghannam, 2009 [22]	USA	16/16	*Pseudomonas* (22), *Klebsiella* (5), *Stenotrophomonas* (3), *Serratia* (2), *E. coli* (1), *Acinetobacter* (1)	Jet nebulizer	Gentamicin 300-400 mg, Amikacin 200-300 mg, Tobramycin 600-900 mg or Colistin 300 mg	9
Kalin, 2012 [23]	Turkey	29/15	*Acinetobacter* (10)	Device not described. Nebulization with oxygen flow of 8 l/min	Colistin 300 mg	9
Kofteridis, 2010 [24]	Greece	43/43	*Acinetobacter* (66), *Klebsiella* (12), *Pseudomonas* (8)	Not described	Colistin 150 mg	9
Korbila, 2010 [29]	Greece	78/43	*Acinetobacter* (92), *Pseudomonas* (17), *Klebsiella* (4)	Ultrasonic nebulizer	Colistin 150 mg	9
Tumbarello, 2013 [31]	Italy	104/104	*Acinetobacter* (128), *Pseudomonas* (52), *Klebsiella* (28)	Jet or ultrasonic nebulizer	Colistin 225 mg	9
**Randomized controlled trials**						Cochrane risk of bias
Hallal, 2007 [25]	USA	5/5	*Pseudomonas* (9), *Acinetobacter* (3), *Staphylococcus* (3)	Jet nebulizer	Tobramycin 600 mg	High
Le Conte, 2000 [26]	France	21/17	*Pseudomonas* (16), *Haemophilus* (6), *Enterobacter* (4), *E. coli* (3), *Klebsiella* (1)	Balloon with a valve connected to the endotracheal tube	Tobramycin 2.5 mg/kg	High
Lu, 2011 [27]	France	20/20	*Pseudomonas* (40)	Vibrating nebulizer	Ceftazidime 120 mg/kg or Amikacin 25 mg/kg	High
Niederman, 2012 [28]	France/Spain/USA	47^a^/22	*Pseudomonas* (24), *E. coli* (14), *Klebsiella* (10), *Acinetobacter* (7)	Vibrating mesh nebulizer	Amikacin 800 mg	Low
Palmer, 2014 [30]	USA	24/18	*Staphylococcus* (18), *Acinetobacter* (12), *Pseudomonas* (9), *Klebsiella* (5), *Enterobacter* (4), Other (9)^b^	Jet nebulizer	Vancomycin 360 mg and/or Gentamicin 240 mg or Amikacin 1200 mg	High
Rattanaumpawan, 2010 [32]	Thailand	51/49	*Acinetobacter* (65), *Pseudomonas* (34), *Klebsiella* (20), *E. coli* (7), *Enterobacter* (3), *Stenotrophomonas* (2)	Jet or ultrasonic nebulizer	Colistin 150 mg	High

1 mg colistin = 13,333 IU. ^a^21 patients randomized to inhaled amikacin 400 mg every 12 hours and 26 patients randomized to inhaled amikacin 400 mg every 24 hours. ^b^ *Proteus* (2), *E. coli* (2), *Stenotrophomonas* (2), *Enterococcus* (1), *Streptococcus* (1), and *Citrobacter* (1). ESBL, extended spectrum beta-lactamase.

The clinical and microbiological cure criteria and adverse events assessed are presented in Table 2. The clinical cure criteria were similar among the studies, with the exception of one study that focused on whether patients were extubated in the 10 days after treatment [26]. One study did not define clinical cure; however, it compared the clinical pulmonary infection score at randomization and at the end of treatment. Because the score was presented as a continuous variable, it was not included in the pooled analysis [30]. Renal toxicity was the most common adverse event assessed (seven out of twelve studies).

**Table 2:** **Clinical and microbiological cure criteria, and adverse events assessment in included studies**

**Study, year**	**Clinical cure criteria**	**Microbiological cure criteria**	**Adverse events assessed**
**Observational studies**			
Doshi, 2013 [21]	Resolution of initial signs and symptoms of infection, including normalization of white blood cell count and temperature, by the end of therapy.	Eradication of the MDR pathogen on subsequent respiratory cultures	NA
Ghannam, 2009 [22]	Improved clinical parameters (fever defervescence, suctioning requirements, symptoms and signs of pneumonia), ventilator parameters and laboratory findings (improved blood gases, normalization of white blood cell count), and/or receding pulmonary infiltrates on a chest radiograph at the end of therapy.	Eradication of causative organisms in patients in whom a follow-up culture was obtained at the end of therapy.	Renal dysfunction (doubling of serum creatinine in patients with pretreatment (baseline) creatinine clearance of ≥30 ml/minute or an increase in creatinine by ≥1 mg/dl at the end of therapy in patients with pretreatment creatinine clearance <30 ml/minute)
Kalin, 2012 [23]	Resolution of symptoms and signs of VAP at the end of the therapy	Eradication of MDR *A. baumannii* on follow-up culture	Renal toxicity (RIFLE criteria)
Kofteridis, 2010 [24]	Resolution of presenting symptoms and signs of infection by the end of colistin treatment	Eradication of the pathogen at the end of antimicrobial therapy or at discharge from ICU	Renal toxicity (serum creatinine value >2 mg/dl; reduction in the calculated creatinine clearance of 50%, compared with the value at the start of treatment; or as a decline in renal function that prompted renal replacement therapy; increase of 150% of the baseline creatinine, a reduction in the calculated creatinine clearance of 50% relative to the value at therapy initiation in patients with pre-existing renal dysfunction), bronchoconstriction, cough, apnea, or chest tightness, and arterial hypoxemia.
Korbila, 2010 [29]	Normalization of temperature and tracheal secretions, together with a return to baseline of the white blood cell count and the C-reactive protein level, and the improvement in chest X-ray appearances, by the end of treatment.	NA	NA
Tumbarello, 2013 [31]	Resolution of all signs and symptoms of pneumonia and improvement or lack of progression of all chest radiograph abnormalities when colistin was discontinued	Disappearance of the infecting bacterium from post-treatment respiratory samples	Acute kidney injury (a greater than twofold increase in serum creatinine or a ≥50% decrease in the glomerular filtration rate or oliguria (output <0.5 ml/kg/hour) for ≥12 hours)
**Randomized controlled trials**			
Hallal, 2007 [25]	Extubation within the study period, improving of MODS, resolution of fever, pulmonary infiltrates and physical signs of pneumonia.	NA	Doubling of the serum creatinine concentration or an increase of creatinine above 2 mg/dl at any time^a^
Le Conte, 2000 [26]	Extubation within 10 days	NA	Respiratory tolerance (described in results section as hypoxemia during nebulization) and evolution of serum creatinine
Lu, 2011 [27]	Reduction of clinical and biological signs of infection, decrease in modified clinical pulmonary infection score below 6, significant lung CT re-aeration, and lower respiratory tract specimens either sterile or with nonsignificant concentrations of *P. aeruginosa*	Eradication of *P. aeruginosa* in a lower respiratory specimens after 8 days of antimicrobial therapy	Bronchospasm, hypoxemia, obstruction of expiratory filter
Niederman, 2012 [28]	Complete or partial resolution of signs and symptoms of pneumonia, improvement or lack of progression of abnormalities on chest X-ray, and no additional intravenous antibiotics since completion of treatment	Confirmed eradication of the original pathogen or presumed eradication in patients with complete or partial resolution of pneumonia	Septic shock, seizures and bronchospasm.
Palmer, 2014 [30]	NA	No growth in culture and no visible organisms seen on Gram-stain of an organism identified at randomization	New resistant to antimicrobial therapy
Rattanaumpawan, 2010 [32]	Complete resolution of all signs and symptoms of pneumonia, and improvement or lack of progression of all abnormalities on the chest radiograph	Eradication or presumed eradication after antimicrobial treatment	Renal impairment (a rise of 2 mg/dl in the serum creatinine level of patients with previously normal renal function or a doubling of the baseline serum creatinine level in patients with pre-existing renal insufficiency), bronchospasm.

^a^This was among the definitions of treatment failure in the trial. CT, computed tomography; MDR, multidrug resistant; MODS, multiple organ dysfunction score; NA, not available; RIFLE, Risk, Injury, Failure, Loss, and End-stage kidney disease; VAP, ventilator-associated pneumonia.

### Study quality

**Figure 2:**
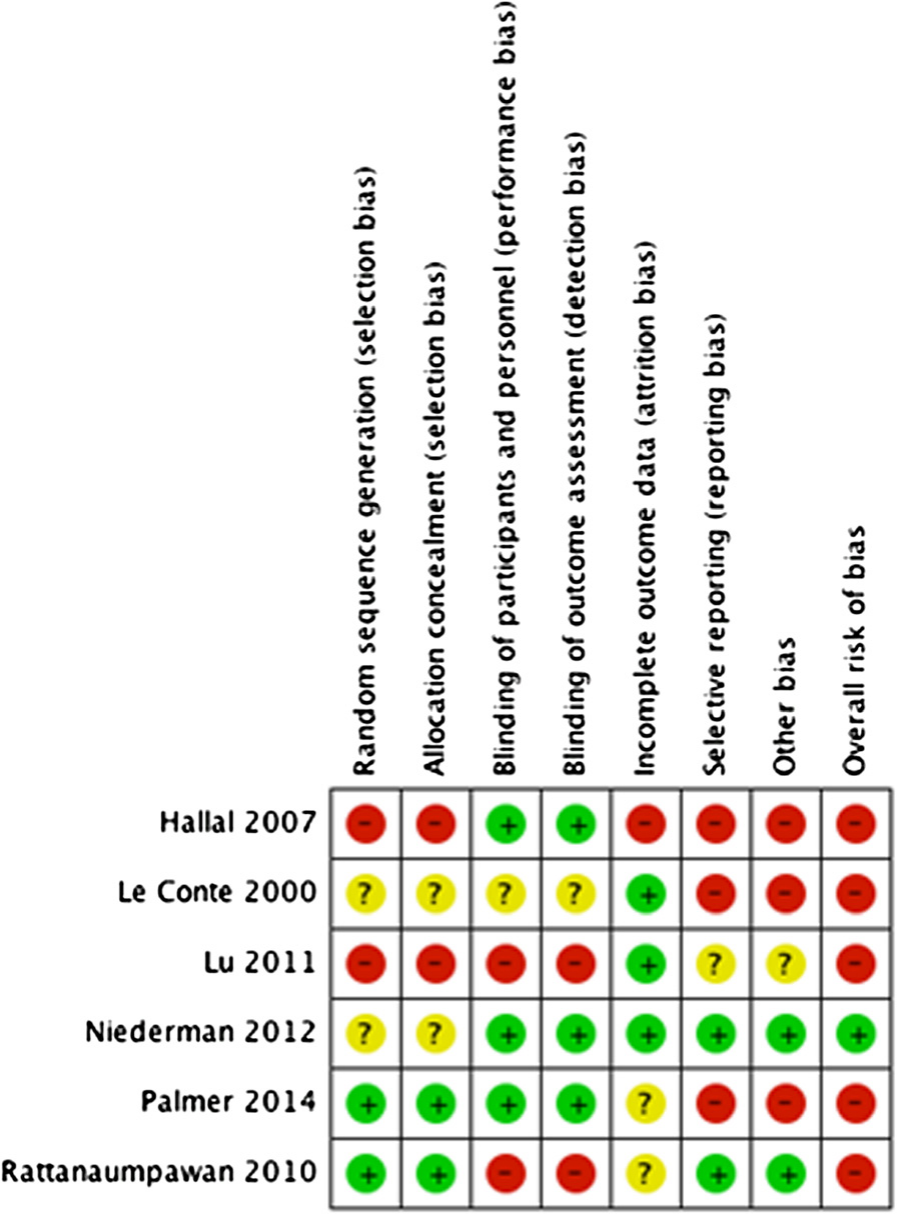
**Risk of bias and a summary are presented as the judgment of the review authors regarding risk of bias for each item included in the study.**

Observational studies were considered high quality according to the Newcastle-Ottawa Scale. Five randomized controlled trials were considered to have a high risk of bias as assessed by the Cochrane risk of bias tool (Table 1). Assessment of each risk of bias is presented in Figure 2.

### Outcomes

**Figure 3:**
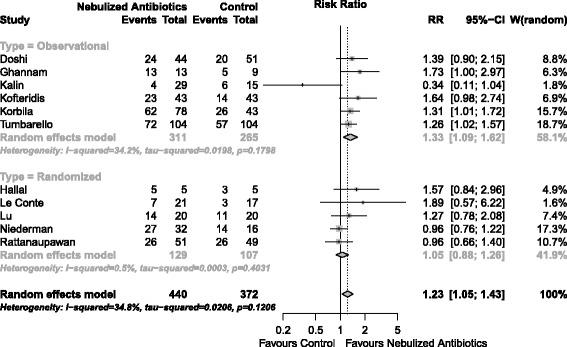
**Forest plot for clinical cure.** *P* for overall effect = 0.009. CI, confidence interval; RR, relative risk.

**Figure 4:**
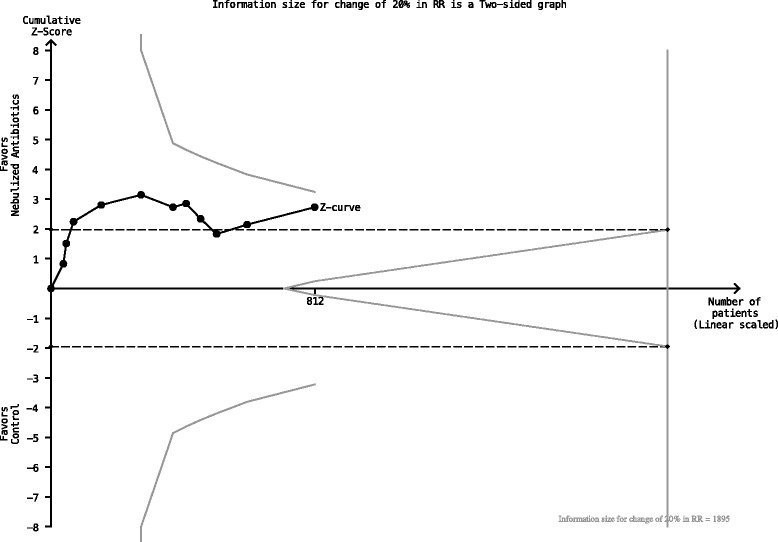
**Trial sequential analysis results.** RR, relative risk.

Clinical cure was assessed in 11 studies totaling 812 patients. Nebulized antibiotics were associated with higher rates of clinical cure (RR = 1.23; 95% CI, 1.05 to 1.43; I^2^ = 34%; D^2^ = 45%) (Figure 3). The Biggertaff-Tweedie model produced similar results (RR = 1.21; 95% CI, 1.19 to 1.22; I^2^ = 34%; D^2^ = 35%). Within the subgroup analysis according to study design (randomized controlled trial versus observational) there was a presence of heterogeneity (Figure 3); however, the difference in the effect of nebulized antibiotics by study design was not significant in the meta-regression (*P* = 0.517). A bubble plot for the meta-regression is shown in Additional file 1 (Figure S1). A funnel plot for the clinical cure analysis is shown in Additional file 1 (Figure S2). The TSA results are shown in Figure 4. Considering the boundaries defined in the methods section, our meta-analysis was insufficiently powered to detect an increase in treatment success with a 5% alpha error limit (information size required 1,895 patients).

**Figure 5:**
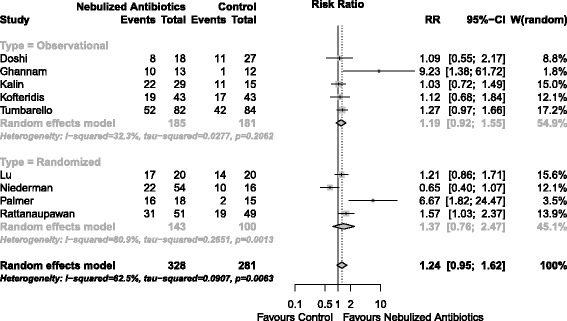
**Forest plot for microbiological cure.** *P* for overall effect = 0.116. CI, confidence interval; RR, relative risk.

Microbiological cure was assessed in eight studies that enrolled a total of 609 patients. The effects of nebulized antibiotics on the microbiological cure were uncertain (RR = 1.24; 95% CI, 0.95 to 1.62; I^2^ = 62.5%) (Figure 5). The funnel plot for the microbiological cure analysis is presented in Additional file 1 (Figure S3) [22,30].

**Figure 6:**
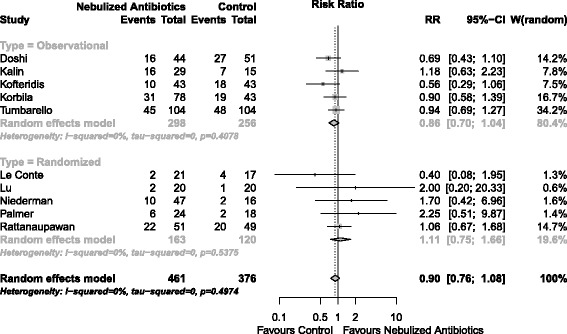
**Forest plot for mortality.** *P* for overall effect = 0.252. CI, confidence interval; RR, relative risk.

Mortality was assessed in 10 studies that enrolled 817 patients. Nebulized antibiotics were not associated with a lower mortality rate compared with the control groups (RR = 0.90; 95% CI, 0.76 to 1.08; I^2^ = 0%) (Figure 6).

The total duration of mechanical ventilation (six studies, 496 patients) and ICU length of stay (six studies, 498 patients) were not affected by nebulized antibiotic use (SMD = −0.10 days; CI 95%, −1.22 to 1.00; I^2^ = 96.5% and SMD = 0.14 days and CI 95% to 0.46-0.73; I^2^ = 89.2%, respectively). Both outcomes had high levels of heterogeneity. The forest plots are shown in Additional file 1 (Figure S4 and Figure S5, respectively).

Renal toxicity was assessed in six studies that enrolled 476 patients. Nebulized antibiotics were not associated with an increased risk of renal toxicity (RR = 1.05; 95% CI, 0.70 to 1.57; I^2^ = 15.6%) (Additional file 1: Figure S6). There were insufficient data available for pooling the risk of other adverse outcomes, such as bronchospasm.

## Discussion

The main finding of the present study was that VAP treatment with nebulized antibiotics might be associated with higher rates of clinical cure. However, a robust analysis aimed at identifying the required information size necessary to detect a significant difference (TSA) demonstrated that our meta-analysis was underpowered for its main clinical outcome. There were no differences regarding the other secondary outcomes, including microbiological cure, mortality or renal toxicity.

Nebulized antibiotics could represent an attractive alternative for VAP treatment, mainly for cases caused by MDR Gram-negative bacteria, because the two most commonly administered intravenous antibiotics (colistin and aminoglycosides) in this scenario do not have adequate lung penetration [33-35]. In contrast, clinical studies in patients with VAP have also confirmed high sputum concentrations of nebulized antibiotics [28]. Despite the good rationale for nebulized antibiotics, there has been a lack of evidence in the current literature to support their use. A recent meta-analysis has suggested nebulized colistin may be effective in the treatment of respiratory infections in ICU patients [36]. However, this meta-analysis included studies of patients with infections other than VAP (such as tracheobronchitis and non-ventilated ICU-acquired pneumonia) and evaluated the role of one specific antibiotic. This meta-analysis concluded colistin has a beneficial effect in the treatment of these respiratory infections, but they did not perform any method to correct the type I error secondary to multiple data analyses [37]. The merits of our meta-analysis are that it has focused specifically on VAP, evaluated a broader range of antibiotics, and included a more robust data analysis. Additionally, we included six trials instead of only one in the previous meta-analysis [36], showing the importance of other antibiotics as inhaled options to treat VAP patients.

Several issues deserve further attention. For example, a deeper understanding of the optimal method for delivering the drug is a crucial next step that could not be assessed in this meta-analysis. There are many factors which may impact on delivery of inhaled particles to lung parenchyma. First, not all types of nebulizers deliver aerosol particles with the same efficiency. Vibrating mesh and ultrasonic nebulizers have appeared to be more efficient in drug delivery than jet nebulizers, because the latter generates aerosol by superimposing a highly turbulent flow to the inspiratory flow coming from the ventilator [11]. This mechanism is associated with lesser deposition of particles in lung parenchyma [38]. Second, spontaneous ventilator modes are associated with high turbulent inspiratory flow and, consequently, delivery of aerosol particles mostly in proximal airways. Ventilator-patient asynchrony may also reduce drug delivery to the lung [39]. Third, ventilator and circuit connections should have smooth inner surfaces and should not have obtuse angles which impair aerosol drug delivery [11]. For the same reason, heat and moisture exchanges should be removed before inhaled therapy [9]. Additionally, antibiotic doses ignoring the inevitable extra-pulmonary deposition may also impact on therapy efficacy [11]. It may be possible that the lack of a clear positive effect found in this meta-analysis could be a consequence of suboptimal delivery of nebulized therapy.

Another important issue is whether inhaled antibiotics should be used as adjunctive therapies or alone for the treatment of VAP. In an experimental model of pneumonia caused by *Pseudomonas*, nebulized colistin provided high drug lung tissue concentrations, whereas intravenous colistin generated undetectable levels in lung tissue [40]. The only included study that directly addressed this question and that was included in this meta-analysis found no differences in the clinical or microbiological cure rates between nebulized monotherapy and intravenous antibiotic regimens [27]. On the other hand, an interesting study from the same group showed that nebulized colistin combined with an intravenous aminoglycoside in the treatment of VAP caused by MDR pathogens is as effective as intravenous combination of a beta-lactam and aminoglycoside or quinolone in the treatment of VAP caused by susceptible pathogens [41].

Additionally, our study highlights some important points regarding the role of nebulized antibiotics in the management of VAP. The available studies are highly heterogeneous and are often associated with high risk of bias. These limitations are reflected in our analysis. We included observational studies and randomized controlled trials, representing a strategy that is sometimes questioned but may have advantages that could outweigh the disadvantages because the addition of more information can aid in clinical decisions [42]. Other strengths of this analysis include the extensive literature review that was performed, which included databases that are typically not searched in systematic reviews. Therefore, the risk of not including a pertinent study was reduced [43]. Finally, the use of a robust statistical analysis with TSA precluded us from making overly optimistic conclusions [37].

## Conclusion

Nebulized antibiotics might be useful for the treatment of VAP; however, the available evidence is of low quality and is highly heterogeneous. The apparent benefit in the clinical cure rate observed in traditional meta-analyses does not persist after TSA. Further high-quality trials in this subject are therefore warranted.

## Key messages

- Nebulized antibiotics may be beneficial for the treatment of VAP.
- However, high heterogeneity and the small number of enrolled patients in the available studies preclude any optimistic conclusions regarding the benefits of nebulized antibiotics.
- High-quality trials analyzing the value of nebulized antibiotics for VAP treatment are warranted.

## Additional file

Additional file 1: Figure S1. Bubble plot for metaregression. No impact of study type in the results was observed. **Figure S2.** Funnel plot for clinical cure. **Figure S3.** Funnel plot for microbiological cure. **Figure S4.** Forest plot for length of mechanical ventilation. *P* for overall effect = 0.864. **Figure S5.** Forest plot for length of ICU stay. *P* for overall effect = 0.651. **Figure S6.** Forest plot for renal injury. *P* for overall effect = 0.823.

## Abbreviations

CI: confidence interval
MDR: multidrug-resistant
RR: relative risk
SMD: standardized mean difference
TSA: trial sequential analysis
VAP: ventilator-associated pneumonia
