# Supplementary material for: Nebulized antibiotics for ventilator-associated pneumonia: a systematic review and meta-analysis
Source: Crit Care. 2015 Apr 7;19(1):150. doi: 10.1186/s13054-015-0868-y (PMC4403838; doi:10.1186/s13054-015-0868-y)

Nebulized Antibiotics for ventilator-associated pneumonia: A systematic review and meta-analysis

Supplementary File

sFigure 1 - Bubble plot for metaregression

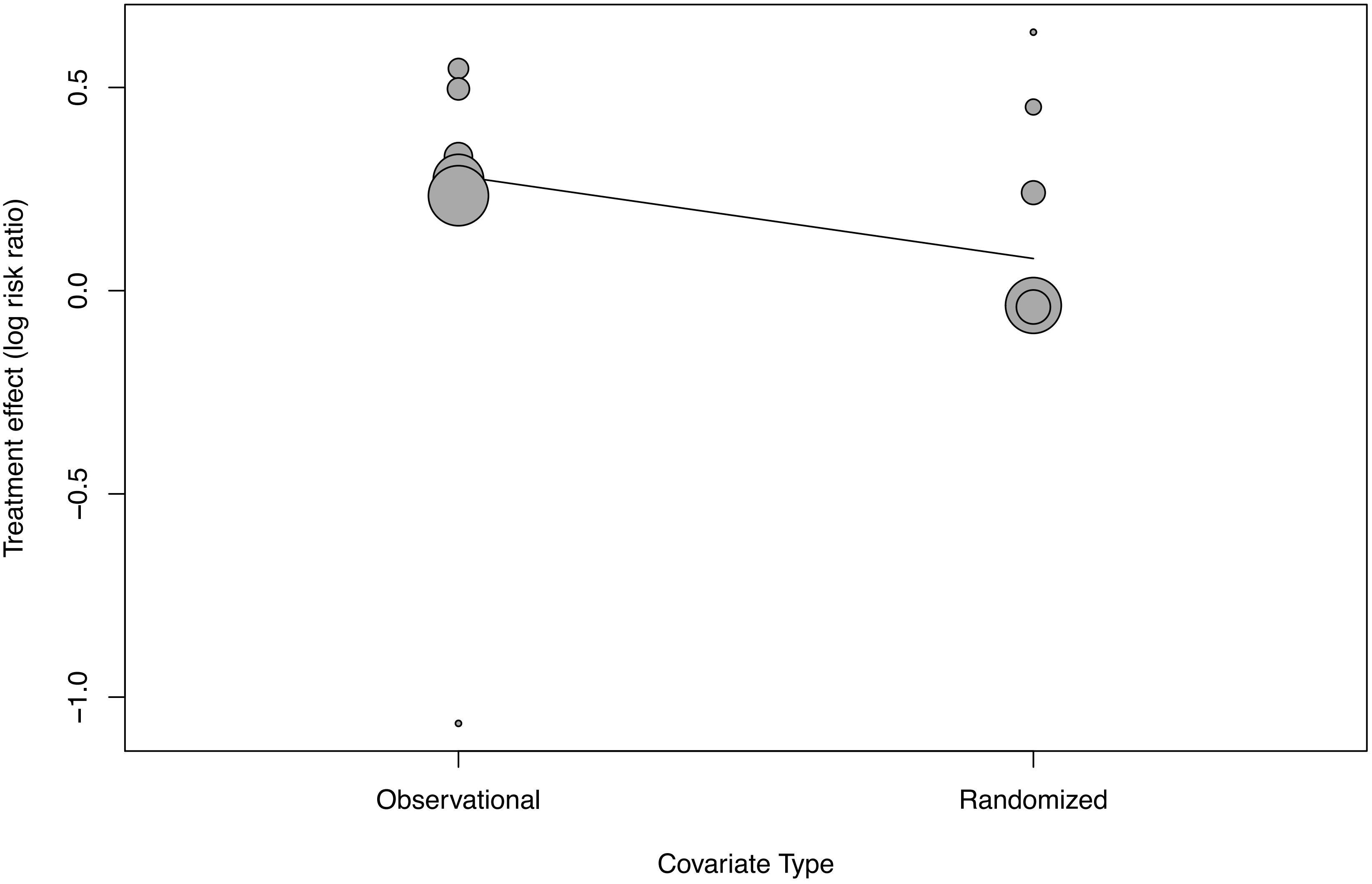

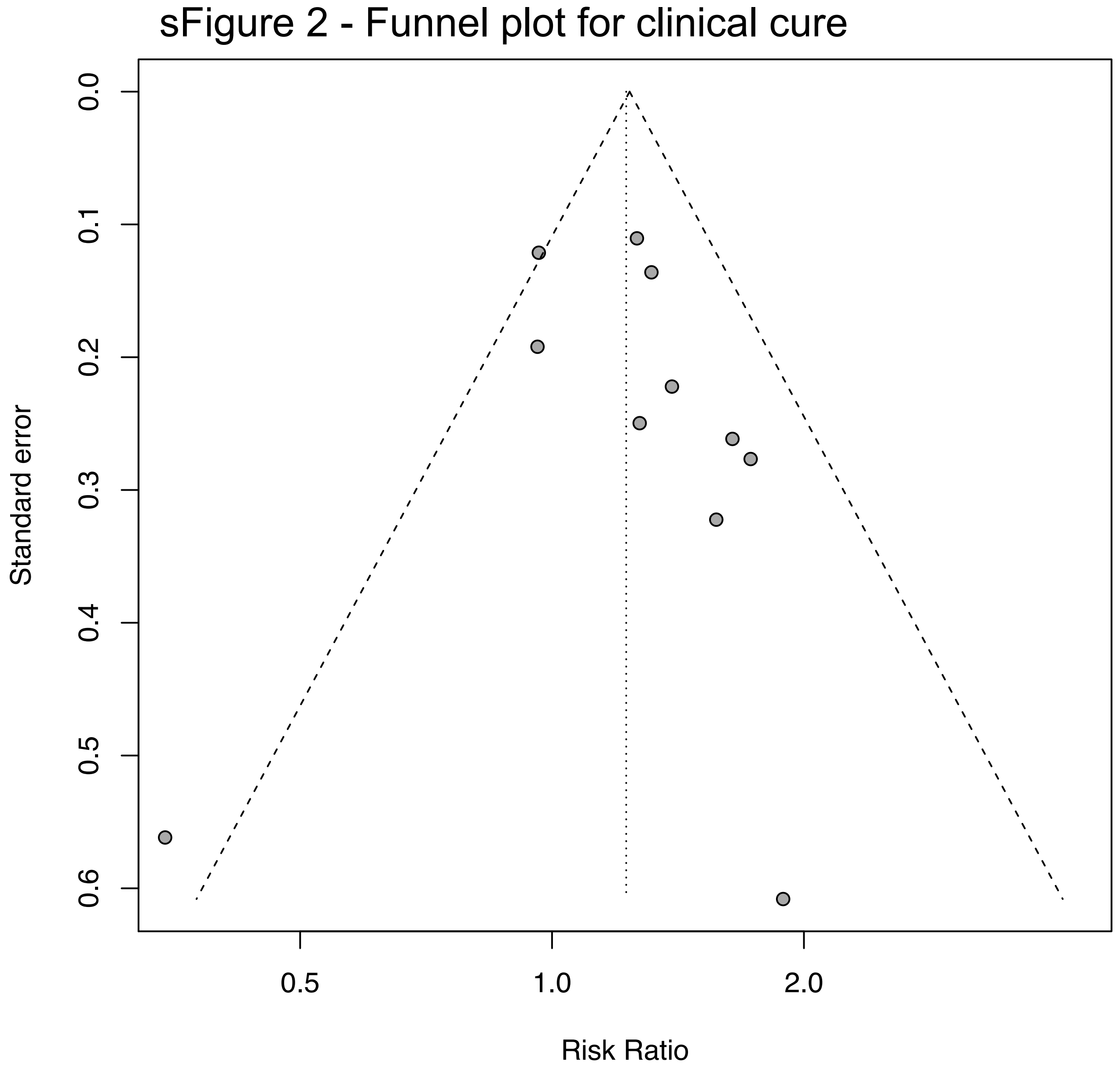

sFigure 2 - Funnel plot for clinical cure
Standard error
0.0
0.1
0.2
0.3
0.4
0.5
0.6
0.5
1.0
2.0
Risk Ratio

sFigure 3 - Funnel plot for microbiological cure

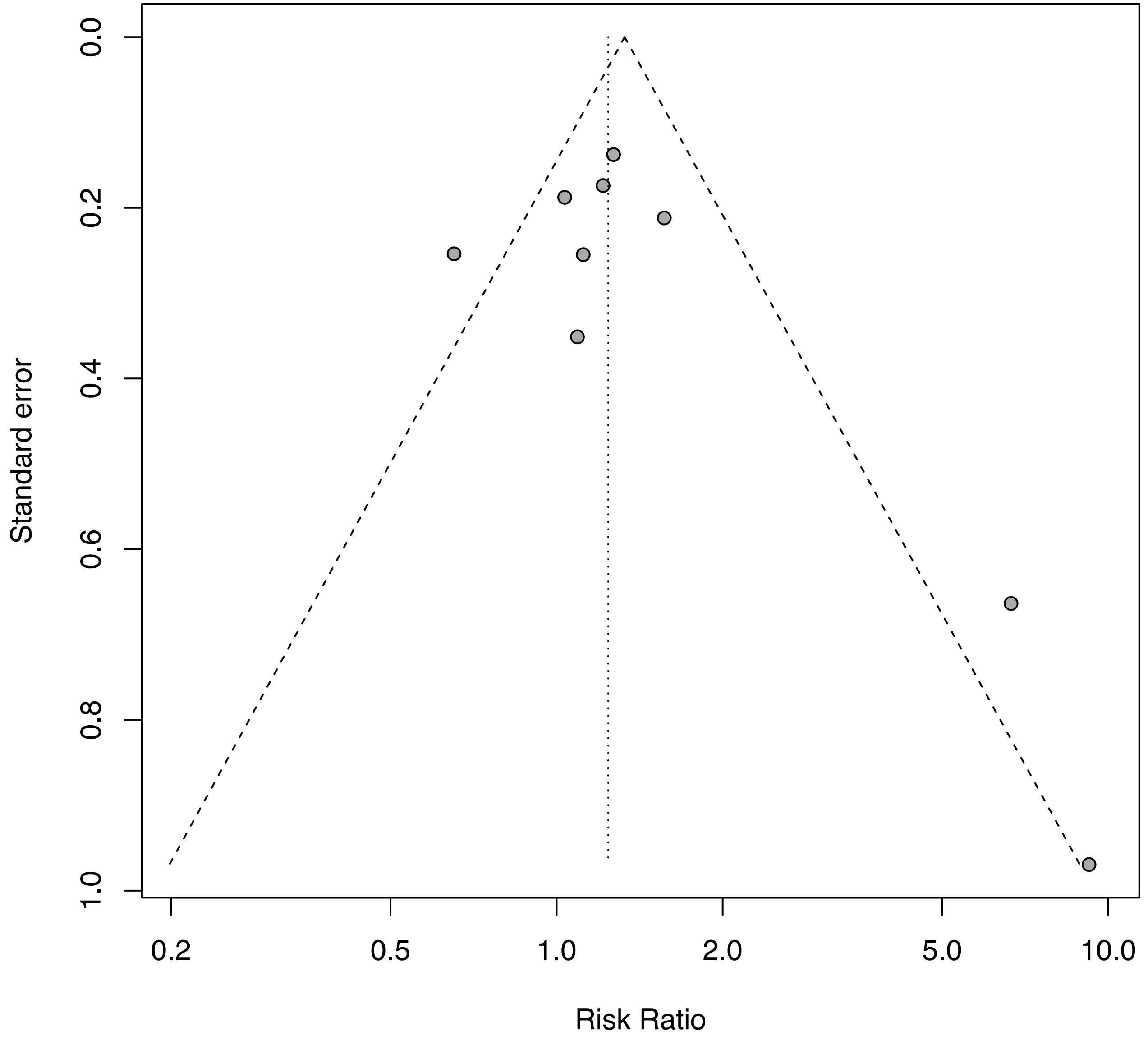

sFigure 4 - Forest plot for Length of Mechanical Ventilation

Study	Nebulized Antibiotics Total	Nebulized Antibiotics Mean	Nebulized Antibiotics SD	Control Total	Control Mean	Control SD	SMD	95%-CI	W(random)
**Type = Observational**									
Doshi, 2013	44	21.65	5.81	51	21.51	8.03	0.02	[−0.38; 0.42]	17.1%
Ghannam, 2009	13	44.25	29.75	12	47.50	32.50	−0.10	[−0.89; 0.68]	16.1%
Kofteridis, 2010	43	15.00	23.50	43	16.50	14.25	−0.08	[−0.50; 0.35]	17.1%
Tumbarello, 2013	104	8.00	1.42	104	12.00	2.17	−2.17	[−2.52; −1.83]	17.2%
**Random effects model**	**204**			**210**			**−0.59**	**[−1.82; 0.63]**	**67.4%**
Heterogeneity: I−squared=96.7%, tau−squared=1.503, p<0.0001									
**Type = Randomized**									
Lu, 2011	20	29.50	4.00	20	20.00	4.50	2.19	[1.39; 2.99]	16.0%
Palmer, 2014	24	12.90	2.10	18	13.50	2.10	−0.28	[−0.89; 0.33]	16.6%
**Random effects model**	**44**			**38**			**0.94**	**[−1.48; 3.36]**	**32.6%**
Heterogeneity: I−squared=95.7%, tau−squared=2.912, p<0.0001									
**Random effects model**	**248**			**248**			**−0.10**	**[−1.20; 1.00]**	**100%**
Heterogeneity: I−squared=96.5%, tau−squared=1.802, p<0.0001									

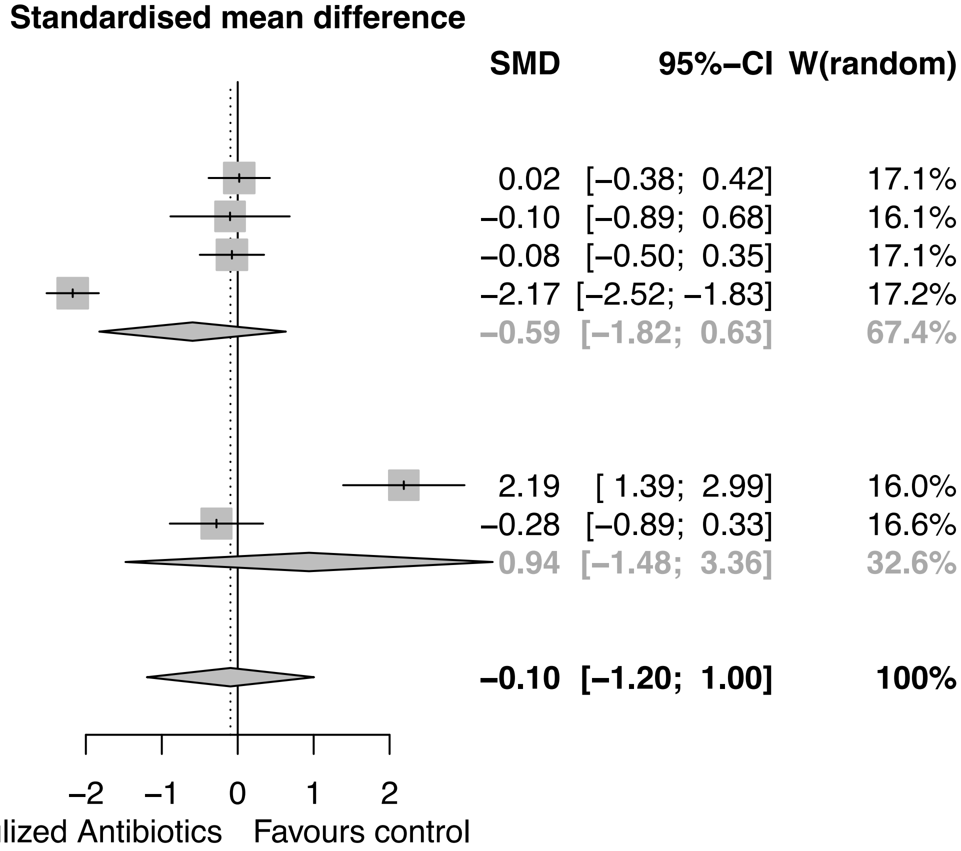

sFigure 5 - Forest plot for Length of ICU Stay

Study	Nebulized Antibiotics Total	Nebulized Antibiotics Mean	Nebulized Antibiotics SD	Control Total	Control Mean	Control SD	SMD	95%–CI	W(random)
**Type = Observational**									
Doshi, 2013	44	24.5	8.44	51	23.0	10.50	0.15	[−0.25; 0.56]	17.9%
Ghannam, 2009	13	58.0	35.00	12	52.5	32.50	0.16	[−0.63; 0.94]	14.5%
Kalin, 2012	29	33.0	34.93	15	36.0	37.63	−0.08	[−0.71; 0.54]	16.0%
Kofteridis, 2010	43	20.5	22.50	43	18.0	18.75	0.12	[−0.30; 0.54]	17.7%
Tumbarello,2013	104	12.0	2.58	104	14.0	2.33	−0.81	[−1.09; −0.53]	18.6%
**Random effects model**	**233**			**225**			**−0.12**	**[−0.61; 0.37]**	**84.8%**
Heterogeneity: I−squared=82.6%, tau−squared=0.2445, p=0.0001									
**Type = Randomized**									
Lu, 2011	20	40.0	6.50	20	30.0	6.50	1.51	[0.80; 2.22]	15.2%
**Random effects model**	**20**			**20**			**1.51**	**[0.80; 2.22]**	**15.2%**
Heterogeneity: not applicable for a single study									
**Random effects model**	**253**			**245**			**0.14**	**[−0.46; 0.73]**	**100%**
Heterogeneity: I−squared=89.2%, tau−squared=0.4748, p<0.0001									

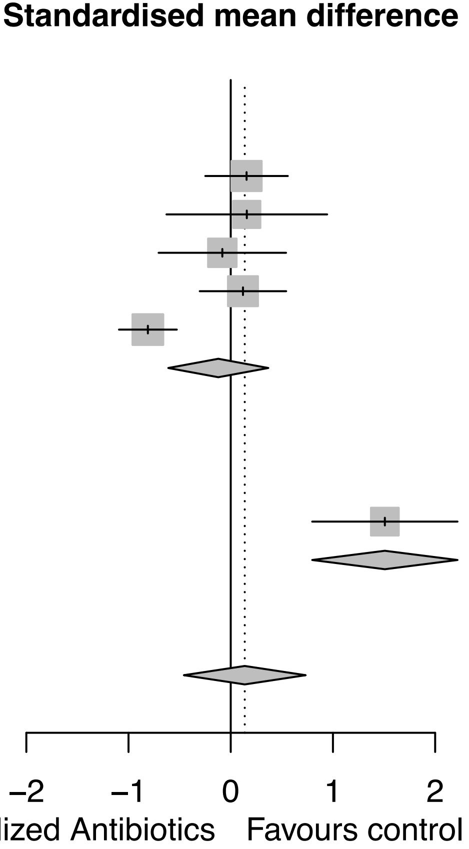

sFigure 6 - Forest plot for Renal Injury

Study	Nebulized Antibiotics Events	Nebulized Antibiotics Total	Control Events	Control Total	RR	95%–CI	W(random)
**Type = Observational**							
Ghannam, 2009	0	16	5	16	0.09	[0.01; 1.52]	2.0%
Kalin, 2012	12	29	3	15	2.07	[0.69; 6.22]	12.0%
Kofteridis, 2010	8	46	8	46	1.00	[0.41; 2.44]	17.2%
Tumbarello, 2013	26	104	23	94	1.02	[0.63; 1.66]	41.4%
**Random effects model**		**195**		**171**	**1.06**	**[0.60; 1.90]**	**72.7%**
Heterogeneity: I–squared=33.2%, tau–squared=0.1174, p=0.213							
**Type = RCT**							
Hallal, 2007	0	5	2	5	0.20	[0.01; 3.28]	2.1%
Rattanaumpawan, 2010	13	51	11	49	1.14	[0.56; 2.29]	25.3%
**Random effects model**		**56**		**54**	**0.82**	**[0.21; 3.15]**	**27.3%**
Heterogeneity: I–squared=29.7%, tau–squared=0.4581, p=0.2329							
**Random effects model**		**251**		**225**	**1.05**	**[0.70; 1.57]**	**100%**
Heterogeneity: I–squared=15.6%, tau–squared=0.0416, p=0.3139							

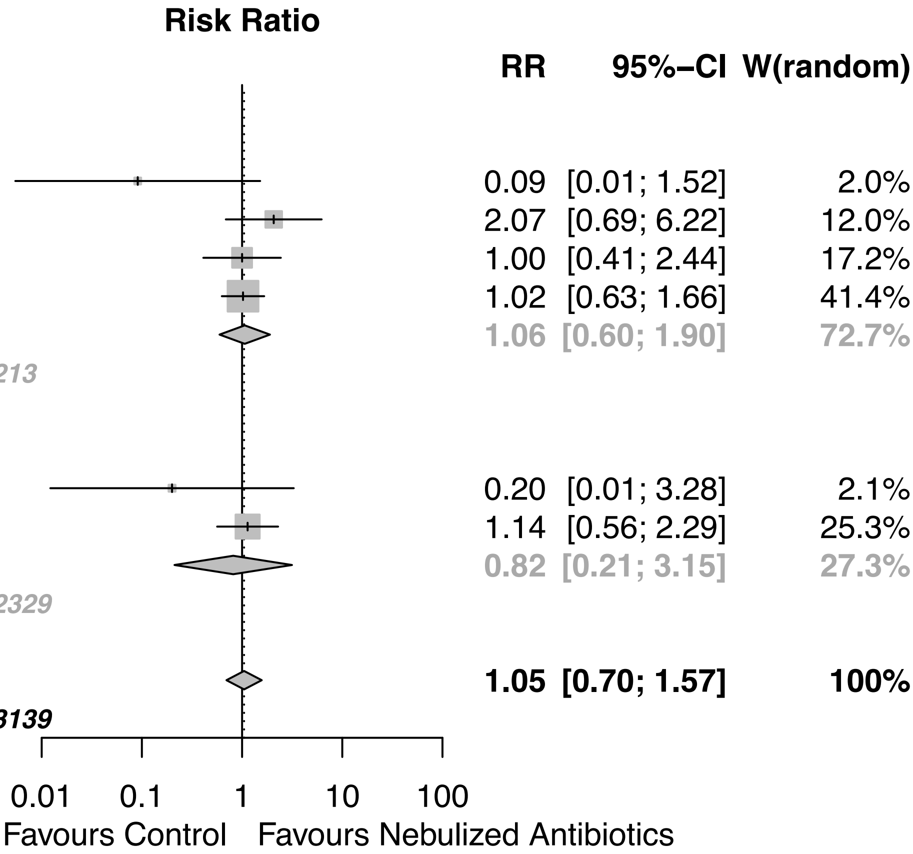

Supplement: Additional file 1: Figure S1. — Bubble plot for metaregression. No impact of study type in the results was observed. Figure S2. Funnel plot for clinical cure. Figure S3. Funnel plot for microbiological cure. Figure S4. Forest plot for length of mechanical ventilation. P for overall effect = 0.864. Figure S5. Forest plot for length of ICU stay. P for overall effect = 0.651. Figure S6. Forest plot for renal injury. P for overall effect = 0.823. [file 13054_2015_868_MOESM1_ESM.pdf]
